# Analysis of electrically evoked compound action potential of the auditory nerve in children with bilateral cochlear implants^☆^

**DOI:** 10.1016/j.bjorl.2014.12.013

**Published:** 2015-12-10

**Authors:** Fernanda Ferreira Caldas, Carolina Costa Cardoso, Monique Antunes de Souza Chelminski Barreto, Marina Santos Teixeira, Anacléia Melo da Silva Hilgenberg, Lucieny Silva Martins Serra, Fayez Bahmad

**Affiliations:** Postgraduate Program in Health Sciences, Universidade de Brasília (UnB), Brasília, DF, Brazil

**Keywords:** Child, Cochlear nerve, Cochlear implant, Evoked potentials, Criança, Nervo coclear, Implante coclear, Potenciais evocados

## Abstract

**Introduction:**

The cochlear implant device has the capacity to measure the electrically evoked compound action potential of the auditory nerve. The neural response telemetry is used in order to measure the electrically evoked compound action potential of the auditory nerve.

**Objective:**

To analyze the electrically evoked compound action potential, through the neural response telemetry, in children with bilateral cochlear implants.

**Methods:**

This is an analytical, prospective, longitudinal, historical cohort study. Six children, aged 1–4 years, with bilateral cochlear implant were assessed at five different intervals during their first year of cochlear implant use.

**Results:**

There were significant differences in follow-up time (*p* = 0.0082) and electrode position (*p* = 0.0019) in the T-NRT measure. There was a significant difference in the interaction between time of follow-up and electrode position (*p* = 0.0143) when measuring the N1-P1 wave amplitude between the three electrodes at each time of follow-up.

**Conclusion:**

The electrically evoked compound action potential measurement using neural response telemetry in children with bilateral cochlear implants during the first year of follow-up was effective in demonstrating the synchronized bilateral development of the peripheral auditory pathways in the studied population.

## Resumo

**Introdução:**

O implante coclear tem a capacidade de medir o potencial de ação composto eletricamente evocado do nervo auditivo (ECAP). Para esta verificação utiliza-se uma medida chamada telemetria de respostas neurais.

**Objetivo:**

Analisar o potencial de ação composto evocado eletricamente, por meio da neurotelemetria de respostas neurais, em crianças usuárias de implante coclear bilateral.

**Método:**

Trata-se de um estudo analítico, prospectivo, de coorte histórica longitudinal. Foram recrutadas seis crianças, com idades entre de 1-4 anos, usuárias de implante coclear bilateral. Estas crianças foram avaliadas em cinco momentos durante o primeiro ano de uso do implante coclear.

**Resultados:**

Houve diferença significativa no tempo de acompanhamento (*p* = 0,0082) e posição do eletrodo (*p* = 0,0019) na medida de T-NRT. Houve diferença significativa na interação entre tempo de acompanhamento e posição do eletrodo (*p* = 0,0143) na medida da amplitude das ondas N1-P1 entre os três eletrodos a cada tempo de acompanhamento.

**Conclusão:**

A mensuração do ECAP por meio da NRT nas crianças com implante coclear bilateral durante o primeiro ano de acompanhamento foi uma medida importante para apresentar o desenvolvimento bilateral da *via* auditiva periférica de forma sincronizada nesta população estudada.

## Introduction

The cochlear implant (CI) device is widely accepted and has been considered one of the most important therapeutic options for patients with severe and/or profound bilateral sensorineural hearing loss, in cases who did not achieve satisfactory auditory perception benefits with the use of individual sound amplification device (ISAD). The CIs have been indicated at progressively younger ages due to advances in early audiological diagnosis and new technologies in the CI devices.^1^

Over the last decades, bilateral CI surgery began to be performed, a procedure that can be performed simultaneously or sequentially. The simultaneous technique is used when the patient receives the two internal components in a single surgical procedure, and the sequential one, when the patient receives the two internal components in different surgical procedures.

Patients who require a CI have been increasingly choosing the bilateral procedure. Some studies have shown that these patients benefit from improved speech in the perception with noise^2^ and improved sound location.^3^, ^4^

The process known as “Programming” or “Mapping” of the CI speech processor is performed at regular intervals postoperatively. The Mapping process aims to determine the appropriate dynamic range of electrical stimulation for each electrode channel. The dynamic range is the difference between the detection perception threshold (T-level) and loudness – maximum comfort (level C).^5^

The measurement of the electrode impedance telemetry can provide an indication of the electrode interface status in the tissues, as well as the appropriate electrode function. Significant changes in these measures can be indicative of changes in the surrounding tissue and/or changes in electrode function. Initial changes in electrode impedance can be expected due to physical changes in the electrode-tissue interface.^6^

The CI has the capacity to measure the electrically evoked compound action potential (ECAP) of the auditory nerve. The system applies an electrical pulse to a specific intracochlear electrode and the evoked neural response is recorded in an adjacent electrode. A measure called Neural Response Telemetry (NRT) is used to assess this potential. The system elicits a valid neural response and robust recordings. These responses are recorded and returned to the programming interface system for clinical analysis.^7^, ^8^

The ECAP provides a relatively direct measurement of the auditory nerve response after electrical stimulation and it is measured in current units (CUs).^9^ The ECAP waveform typically consists of an initial negative peak followed by a positive peak, called N1 and P1, respectively.^7^, ^8^

The NRT threshold (T-NRT) is defined as the smallest amount of electric current that can evoke these physiological responses. Studies have shown that the T-NRT measured intraoperatively or at postoperative intervals can be correlated with the psychophysical detection of the threshold (T level) and the maximum level of comfort (C level) in patients with CIs. The amplitude of the response (measured between N1 and P1) varies with increasing stimulus intensity and it is measured in millivolts (μV).^10^, ^11^, ^12^

Considering the abovementioned facts, this study aims to analyze the ECAP through NRT in children who received bilateral cochlear implants. The ECAP will be analyzed in relation to the T-NRT visual threshold and the amplitude of N1-P1 peak during the first year of CI use.

## Methods

The study was approved by the Research Ethics Committee (REC) of Faculdade de Ciências da Saúde, Opinion number 571,432/2014. The participants’ parents or guardians signed the free and informed consent form (FICF) accepting their participation in the study.

This is an analytical, prospective, longitudinal contemporary cohort study.

## Sample

The study included six children, five females and one male. Ages ranged from 1 to 4 years. The children had congenital hearing loss and were Nucleus^®^ bilateral cochlear implant users (Cochlear Corporation), having been submitted to simultaneous surgery and total electrode insertion. Data were analyzed between January (2012) and March (2014). Participants with unilateral cochlear implant, auditory neuropathy spectrum disorder (ANSD), partial insertion of the electrode array and those submitted to the sequential technique during internal component surgery was excluded.

### Equipment

A speech processor, external antenna (coil) with magnet, connector cable between speech processor and the external antenna, programming interface – POD (Programming POD), and the computer used to send and receive neural information were used to evaluate impedance telemetry and neural responses.

### Procedure

The impedance telemetry and neural response recordings were collected through an AutoNRT system, using the software Custom Sound EP 3.2 version to perform intraoperative measurements and Custom Sound 3.2 for postoperative measurements.

The impedance telemetry and NRT were first performed in the operating room, after the internal component insertion and while the child was still anesthetized, and these procedures also continued to be carried out in the postoperative period. During the intraoperative period, the neural responses were recorded in the 22 electrodes; however, for the analysis in this study only the electrodes E1, E11 and E22 were used. Postoperatively, the participants were evaluated at five moments (first, third, sixth and twelfth months) after surgery, and the responses were recorded in the electrodes E1, E11 and E22.

The impedances were measured in the MP1 monopolar, MP2 monopolar, MP1+2 monopolar and Common Ground (CG) modes. Values were considered normal when between 0.7 kΩ and 30 kΩ. Electrodes with electrical problems such as short circuits – “short” (<0.7 kΩ) and open circuits – “open” (>30 kΩ) were not selected.

The parameters for ECAP recording were: interpulse interval of 400 μs, stimulation rate of 80 Hz with series of 25 μs of pulse width, the number of presentations varied between 100 and 200 pulses per second for amplifier gains in, respectively, 50 dB, the window for recording was 1600 μs. The current level of the masking noise was fixed at 10 units above the stimulation level.

All participants received the same parameters in CI programming in the postoperative period. The parameters used were in accordance with the standardization of Cochlear Corporation: ACE (advanced combination encoder) speech coding strategy, mode of stimulation MP1 + 2, stimulation rate of 900 pulses per second per channel, eight maximas and pulse width of 25 μs.

For each electrode, the characteristics of the T-NRT threshold and peak amplitude of N1-P1 waves were compared between the returns. These measurements were separately compared for: the right and left ear, the intra and postoperative periods and electrode position. Electrode 01 (E01) was called basal, electrode 11 (E11) was called middle and electrode 22 (E22) was called apical. These nominations are in accordance with their positions in the cochlear region.

The “activation” of the CI electrodes occurred within one month after the intraoperative period, which would be the first time the device was activated, thus sending the electrical signal to the auditory nerve through the 22 intracochlear electrodes; after this period, the postoperative measurements were started.

From each measured electrode, only the valid responses were selected, that is, the NRT responses had to be identifiable, even though it is known that responses are increasingly less robust in the electrical threshold.

The answers were analyzed through the automatic software function. The corresponding recording location was chosen for the electrodes to be analyzed in basal, middle and apical form (E1, E11 and E22 electrodes). When this was not possible, that is, when these electrodes did not respond or when the children showed some discomfort, the closest electrodes’ responses were not considered.

### Statistical analysis

The results of this study were analyzed by a mixed-effect model of analysis of variance for repeated measures. Once the measurements of each individual were obtained in both ears, in the three assessed electrodes and during follow-up, a factorial design structure was used in the model.

When the global *p*-value of any factor was less than 0.05, a Bonferroni correction was used to adjust the multiple tested comparisons. Analyses were performed using the SAS 9.3 software.

## Results

Table 1 shows the participants’ characterization. All children had profound sensorineural hearing loss, of idiopathic etiology.

**Table 1:** Characteristics of participants.

Participants	Etiology	Type and degree of hearing loss	Age at CI surgery (months)	Cochlear implant processor	Time of CI use (months)
1	Idiopathic	Profound sensorineural	14	CP810	5
2	Idiopathic	Profound sensorineural	22	CP810	12
3	Idiopathic	Profound sensorineural	24	CP810	16
4	Idiopathic	Profound sensorineural	24	CP810	16
5	Idiopathic	Profound sensorineural	27	CP810	14
6	Idiopathic	Profound sensorineural	30	CP810	25

CI, cochlear implant.

Table 2 shows the mean age of participants at the CI surgery and the time of CI use (months). The mean age at surgery was 23.50 months (standard deviation 5.43) and the time of cochlear implant use was 14.67 months (standard deviation 6.50).

**Table 2:** Mean age (months) at surgery and time of CI use.

Variable	Mean	Standard deviation
Age at CI surgery	23.50	5.43
Time of CI use	14.67	6.50

CI, cochlear implant.

The results showed the ECAP response measured by NRT. After that, the responses of two measures used in this study, the visual T-NRT and the amplitude of N1-P1 waves, will be shown.

### T-NRT visual measurement

Initially, we adjusted the model of mixed-effect analysis of variance with repeated measures. We evaluated the factors: ear (right and left), time of follow-up between intraoperative and postoperative periods and position of electrodes in the cochlea. All these measures were also correlated with each other. The results are shown in Table 3.

**Table 3:** Analysis of variance chart.

Factors	*F* value	*p*-value
Ear	3.50	0.1202
Time	4.80	0.0082^a^
Electrode	12.49	0.0019^a^
Ear × time	1.31	0.3035
Ear × electrode	0.10	0.9023
Time × electrode	0.66	0.7171
Ear × time × electrode	0.55	0.8086

a *p*-value with Bonferroni correction. Statistically significant difference (*p* < 0.05).

Once we detected significant differences in the time of follow-up (*p* = 0.0082) and electrode position (*p* = 0.0019), the analysis was continued by comparing the mean T-NRT values, separately for each factor, using multiple comparisons two by two between the levels of each factor with Bonferroni correction.

Table 4 shows the mean values and standard deviations for the factors: ear, position of the electrodes in the cochlea and time of follow-up between intraoperative and postoperative periods. These factors were assessed separately.

**Table 4:** Mean (current units) and standard deviation per factor.

Ear	Time	Electrode	Mean	Standard deviation
Right			174.79	2.51
Left			169.99	2.76
		E01	180.49	2.88
		E11	182.14	2.73
		E22	156.15	2.76
	Intra-op		188.64	3.77
	1st m postop		166.03	4.03
	3rd m postop		168.12	3.53
	6th m postop		167.68	3.62
	12th m postop		168.07	4.47

Intra-op, intraoperative; postop, postoperative; m, months.

**Figure 1:**
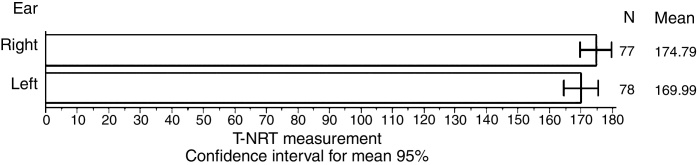
Mean T-NRT values for each ear separately.

Fig. 1 shows the mean values of the T-NRT threshold for each ear, separately.

**Figure 2:**
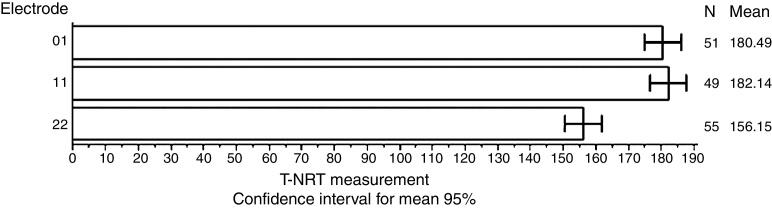
Mean T-NRT (current units) values in each electrode.

Fig. 2 shows the mean values for T-NRT measured in each electrode, separately. To analyze these results, three electrodes were assessed in each ear, at five different times, during the first year of CI use.

Thus, a total of 180 measurements were performed (60 for each electrode). The results obtained were 51 measurements in basal electrodes, 49 in medial electrodes and 55 in apical electrodes, totaling 155 measurements. It is noteworthy that participant 1 was assessed only up to the first three months of CI use, because she had used the device for only 5 months, and thus 12 measurements were lost (2 in electrode E01, 2 in electrode E011 and 2 in electrode E22). Excluding this measurement in this participant, 168 measurements remained. Of this number, 13 measurements showed absence in the NRT measurement.

Table 5 shows multiple comparisons for the electrode factor and *p*-values with Bonferroni correction.

**Table 5:** Multiple comparisons for the electrode factor.

Multiple comparisons – electrode factor	*p*-value^a^
E01 × E01	–
E01 × E11	1.0000
E01 × E22	0.0060^a^
E11 × E01	1.0000
E11 × E11	–
E11 × E22	0.0036^a^
E22 × E01	0.0060^a^
E22 × E11	0.0036^a^
E22 × E22	

a *p*-value with Bonferroni correction. Statistically significant difference (*p* < 0.05).

The mean value of T-NRT in electrode E01 was significantly higher than in electrode E22 (*p* = 0.0075); the mean value of T-NRT in electrode 11 was significantly higher than in electrode 22 (*p* = 0.0035), whereas the mean values of T-NRT did not differ significantly between electrode E01 and electrode E11.

Table 6 shows multiple comparisons for the time factor (*p*-values) with Bonferroni correction.

**Table 6:** Multiple comparisons for the time of follow-up factor.

Time factor	Multiple comparisons – time factor				
	Intra-op	1st m postop	3rd m postop	6th m postop	12th m postop
Intra-op	–	0.0238^a^	0.0203^a^	0.0970	0.1056
1st m postop	0.0238^a^	–	1.0000	1.0000	1.0000
3rd m postop	0.0203^a^	1.0000	–	1.0000	1.0000
6th m postop	0.0970	1.0000	1.0000	–	1.0000
12th m postop	0.1056	1.0000	1.0000	1.0000	–

Intra-op, intraoperative; postop, post-operative; m, months.

a *p*-value with Bonferroni correction. Statistically significant difference (*p* < 0.05).

The mean value of T-NRT intraoperatively was significantly higher than the mean T-NRT values in the first (*p* = 0.0238) and third (*p* = 0.0203) months of follow-up. There is no significant difference in other comparisons between times of follow-up (6th month and 12th month).

**Figure 3:**
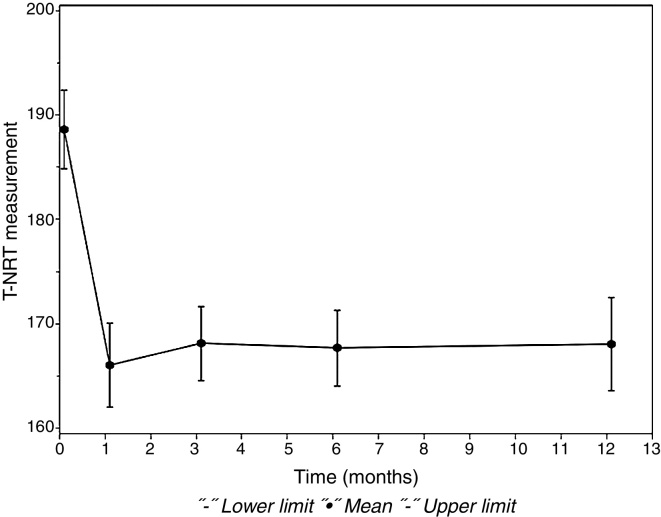
Difference in T-NRT (current units) between the intraoperative and the postoperative follow-up periods.

Fig. 3 shows this significant difference of *p*-value between the intraoperative period and the first and third months of follow-up. At 6 months and 12 months of follow-up, the measured T-NRT values were not significant.

### Measurement of the N1-P1 wave amplitude

In the results of the N1-P1 wave amplitude measurements, initially the adjustment of the model of mixed-effect analysis of variance with repeated measures was carried out. We evaluated the factors: ear (right and left), time of follow-up between intraoperative and postoperative periods and position of electrodes in the cochlea. All these measures were also correlated with each other. The results are shown in Table 7.

**Table 7:** Analysis of variance chart.

Factors	*F* value	*p*-value
Ear	1.75	0.2429
Time	3.57	0.0259^a^
Electrode	1.62	0.2458
Ear × time	0.35	0.8421
Ear × electrode	0.90	0.4387
Time × electrode	3.07	0.0143^a^
Ear × time × electrode	0.76	0.6380

a *p*-value with Bonferroni correction. Statistically significant difference (*p* < 0.05).

Once a significant difference was detected in the interaction between time of follow-up and electrode position (*p* = 0.0143), the analysis was continued by comparing the mean values of N1-P1 wave amplitude, between the three electrodes at each time of follow-up, with the use of multiple comparisons two by two between the levels of each electrode at each time, with Bonferroni correction.

Table 8 shows the mean values and the respective standard deviations for each electrode and time combination.

**Table 8:** Mean and standard deviation for each combination of time and electrode in the measurement of the amplitude of N1-P1 waves (μV).

Time	Electrode	Mean	Standard deviation
Intra-op	E01	12.7675	9.8181
Intra-op	E11	20.6550	9.8181
Intra-op	E22	17.3858	9.8181
1rd m postop	E01	23.6229	10.0431
1rd m postop	E11	22.6594	9.9920
1rd m postop	E22	24.1875	9.8181
3rd m postop	E01	17.2277	9.9069
3rd m postop	E11	15.7465	10.0292
3rd m postop	E22	26.2308	9.8181
6th m postop	E01	34.2599	10.8887
6th m postop	E11	31.0511	11.2435
6th m postop	E22	35.3715	10.8859
12th m postop	E01	45.7199	10.8762
12th m postop	E11	45.7500	10.7552
12th m postop	E22	59.2800	10.7552

Intra-op, intraoperative; postop, postoperative; m, months.

Table 9 shows multiple comparisons between the electrodes at each time, adjusted by Bonferroni correction factor.

**Table 9:** Multiple comparisons between the electrodes at each follow-up of the measurement of amplitude of N1-P1 waves (μV).

Multiple comparisons	*t* value	*p*-value^a^
Intra-op – E01 × E11	−3.12	0.0645
Intra-op – E01 × E22	−1.53	1.0000
Intra-op – E11 × E22	1.59	1.0000
1st m post-op – E01 × E11	0.37	1.0000
1st m post-op – E01 × E22	0.23	1.0000
1st m post-op – E11 × E22	−0.16	1.0000
3rd m post-op – E01 × E11	1.03	1.0000
3rd m post-op – E01 × E22	−2.49	0.2925
3rd m post-op – E11 × E22	−3.46	0.0285^a^
6th m post-op – E01 × E11	0.65	1.0000
6th m post-op – E01 × E22	−0.25	1.0000
6th m post-op – E11 × E22	−0.87	1.0000
12th m post-op – E01 × E11	1.06	1.0000
12th m post-op – E01 × E22	−0.33	1.0000
12th m post-op – E11 × E22	−1.43	1.0000

Intra-op, intraoperative; post-op, postoperative; m, months.

a *p*-value with Bonferroni correction. Statistically significant difference (*p* < 0.05).

In the third month of follow-up in the postoperative period, the mean N1-P1 wave amplitude (μV) value in electrode E11 was significantly lower than the mean N1-P1 wave amplitude value in electrode E22 (*p* = 0.0285).

**Figure 4:**
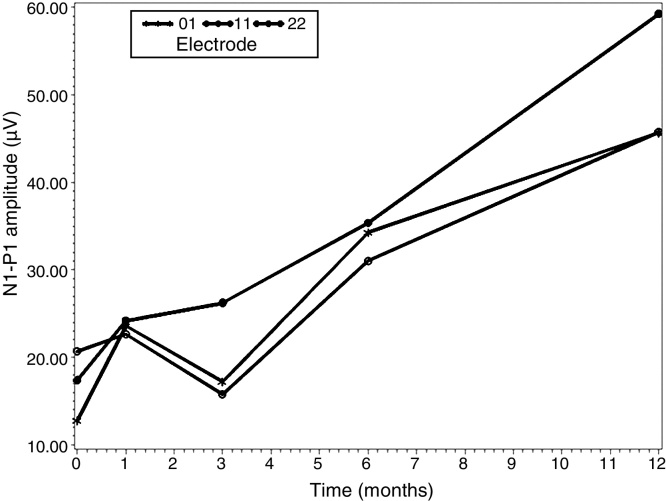
Mean value of N1-P1 wave amplitude (μV) in the third month of follow-up between E11 and E22.

Fig. 4 shows this significance of the *p*-value in the 3rd month of follow-up between E11 and E22.

## Discussion

While measuring impedance telemetry during the 12 months, normal values were observed for all the assessed electrodes. Thus, it was not necessary to exclude any electrode for the measurements used in this study. These results also showed integrity of the cochlear implant internal component. These data agree with the study by Hughes et al.,^6^ who reported that this measure provides an appropriate indication about the electrode interface status in tissue, as well as the appropriate function of the electrode.

### T-NRT visual threshold

In Fig. 1, there was no significant difference between the ears (*p* = 0.1202) in T-NRT measure and in the measured N1-P1 wave amplitude (*p* = 0.2429) (Table 7). These findings allow us to infer that bilateral CI with simultaneous surgical technique, for this study population, was effective for synchronized neural stimulation in both ears. The stimulation provided by a cochlear implant is single, and a large electric pulse can promote greater stimulation on neurons for the auditory nerve to respond to the stimulus in a highly synchronized manner, allowing benefits for children with severe hearing loss.^13^

Gordon et al.^14^ performed a study with EABR (Electrically Evoked Auditory Brainstem Response) and concluded that the reduction of latencies in the waves may reflect an increase in neural synchrony or a more rapid nerve conduction in the brain stem promoted by bilateral stimulation *versus* unilateral stimulation.

The ECAP through the NRT measure only peripherally assesses the auditory pathway (auditory nerve), but it is important to emphasize that this electrical stimulation triggered in the auditory pathway is one of the precursors to make auditory information effectively arrive at the auditory cortex.^10^

Fig. 2 shows the mean values for T-NRT measurement in each electrode separately. Postoperatively, absences were observed in the recordings of this measure, which can be explained by some discomfort during the procedure (4 measurements) and the remaining by the absence of neural response *per se* (9 measurements, that is, 4 in electrode E01, 4 in electrode E11 and 1 in electrode E22). These data are similar to other data from literature.^15^ Lai et al.^16^ state that, over time, the trend of NRT levels improve and generally become stable. These responses to electrical stimulation of electrodes show variations according to their position within the cochlea and the density and integrity of the neural population.^17^

In this study, the mean T-NRT measurement (Table 4) during the intraoperative period was 188.64 CUs, whereas in the postoperative period it was 166.03 CUs in the first month; 168.12 CUs in the third month; 167.68 CUs in the sixth month, and 171.50 CUs in the 12th month. The mean value of T-NRT in the intraoperative period was significantly higher than the mean T-NRT values in the first (*p* = 0.0238) and third (*p* = 0.0203) months of follow-up. There is no significant difference in other comparisons between periods (6, 9, 12 months).

However, in the studies of Tanamati et al.,^18^ Hughes et al.,^6^ Thai Van et al.,^10^ and Muhaimeed et al.^19^ there were no statistically significant differences in the T-NRT threshold during the first year of device programming. Lai et al.^16^ also found no significant difference in this measure in the first 15 months of device use. In these literature findings, the CI surgery was performed with participants from different age groups. Only in the study of Tanamati et al.^18^ a younger age at surgery was observed, before 3 years, but with no statistical significance. The age at CI surgery (23.50 months) and the bilateral stimulation of the hearing nerve can justify the significant finding during the first three months in our population.

To justify the absence of significant correlation after 3 months of CI use, it should be considered that the NRT measurement only allows an investigation of the neural responsiveness capability in the peripheral auditory system. Thus, one can infer that the first three months were essential for the stimulation of the auditory nerve in the children followed in this study and that the neural response remained in the presence of electrical stimulation after this period. The NRT measurement cannot guarantee that the cognitive mechanisms involved in the auditory perception are activated.^10^

Table 5 shows that the mean value of T-NRT in electrode E01 was significantly higher than in electrode E22 (*p* = 0.0075); the mean value of T-NRT in electrode E11 was significantly higher than in electrode E22 (*p* = 0.0035), and the mean values of T-NRT did not differ significantly between electrodes E01 and E11.

These data are similar to those reported by Dees et al.,^20^ Vlahovic et al.^21^ and Brown et al.,^22^ who observed a significant effect between the apical and basal electrodes. Considering the literature findings correlated to the present study data, we can assume that chronic stimulation of the low-frequency cochlear region during the preoperative period may have contributed to improve neuronal survival and/or improve neuronal synchrony of peripheral neurons in the apical portion of the cochlea, or that performing hearing rehabilitation preoperatively, and the activation and development of the central auditory pathways contribute to the functional maintenance of spiral ganglion cells in the apical portion of the cochlea.^21^

### N1-P1 peak amplitude

In Table 7, the time of CI use and the electrode position were correlated, and there was a significant difference (*p* = 0.0143) in the N1-P1 amplitude with these factors. Gordon et al.^13^ also found significance in the increase of the N1 peak amplitude in the ECAP and in the V wave in EABR, during the time of CI use. Tanamati et al.^18^ evaluated the first year of CI use and found that in all electrodes there was an increase in the N1 peak amplitude between the second and third returns.

According to these results of the N1-P1 peak amplitude in ECAP, it can be assumed that changes in the ECAP amplitude suggest an improvement in neural synchrony in the primary auditory nerve during the time of CI use. A greater synchronization could, in theory, be due to a change in the way the stimulation reaches and activates the primary nerve fibers and/or a reduced variation in the onset of neuronal response times. The increase in wave amplitude suggests, therefore, that the continuous stimulation activates more neurons, and thus a greater number can be recruited to establish a response with synchrony.^13^

Table 9 and Fig. 4 show that, in the third month of postoperative follow-up, the mean value of N1-PI wave amplitude (μV) in electrode E11 was significantly lower than the mean N1-P1 wave amplitude in electrode E22 (*p* = 0.0285). There were no significant differences for other comparisons between the electrodes at each time period.

Gordon et al.,^23^ Hughles et al.^6^ and Vlahovic et al.^21^ also found higher amplitude of ECAP in the apical electrodes than in the medial and basal electrodes. These results suggest that electrical stimulation of the basal electrodes may involve a smaller number of spiral ganglion cells, when compared to stimulation in the intermediate or apical electrodes.^23^ The amplitudes reflect the sum of the neuron activities, that is, the number of neurons that respond to a stimulus, which in turn indicate better preservation of neuronal function.^21^

Considering the data shown in this study, we observed that the auditory pathways in implanted children become more efficient during the first year of cochlear implant use, probably because the groups of nerves respond faster and with a greater degree of synchrony. These activity-dependent processes are probably due to increased synaptic networks, and perhaps to an increase in myelination.^13^

## Conclusion

Based on the analysis of neural responses in six children evaluated in the first year of use of bilateral cochlear implants, we concluded that the measurement of ECAP by NRT was an important measure to assess the bilateral development of the peripheral auditory pathway in a synchronized manner in this assessed population.

## Conflicts of interest

The authors declare no conflicts of interest.
